# Analysis of eplerenone in the FDA adverse event reporting system (FAERS) database: a focus on overall patient population and gender-specific subgroups

**DOI:** 10.3389/fphar.2024.1417951

**Published:** 2024-07-17

**Authors:** Xin Guan, Yusi Yang, Xinru Li, Yue Feng, Jizhen Li, Xuewen Li

**Affiliations:** ^1^ Department of Cardiology, Third Hospital of Shanxi Medical University, Shanxi Bethune Hospital, Shanxi Academy of Medical Sciences Tongji Shanxi Hospital, Taiyuan, China; ^2^ Second School of Clinical Medicine, Shanxi Medical University, Taiyuan, China

**Keywords:** eplerenone, FAERS database, safety, adverse event, adverse drug reaction

## Abstract

**Introduction:** Eplerenone is approved for the treatment of hypertension as well as symptomatic heart failure with reduced ejection fraction (HFrEF) following an acute myocardial infarction. However, the adverse events (AEs) have not been systematically analyzed. The aim of this study was to identify adverse drug reactions (ADRs) related to eplerenone using the FDA Adverse Event Reporting System (FAERS) database. By identifying previously unreported AEs, the study could potentially contribute to updating the drug’s label.

**Methods:** In order to find significant AEs, four algorithms, including Reporting Odds Ratio (ROR), Proportional Reporting Ratio (PRR), Bayesian Confidence Propagation Neural Network (BCPNN) and Empirical Bayesian Geometric Mean (EBGM), were used to analyze the signal strength of the ADRs connected to eplerenone that were gathered from the FAERS database over the previous 20 years.

**Results:** From 2004Q1 to 2023Q4, a total of 20, 629, 811 reported cases were gathered from the FAERS database for this study. After processing the data and filtering, 1,874 case reports were analyzed. Of these cases, 1,070 AEs were identified, 128 of which were eplerenone-related ADRs. We investigated the occurrence of ADRs induced by eplerenone in 27 organ systems. Our study showed that the AEs listed in the medication’s package insert correspond with those listed in the literature, including hyperkalemia and increased creatinine. Additionally, the prescription label for eplerenone does not include all system organ class (SOC) terms, like Vascular disorders, hepatobiliary Disorders, etc.

**Discussion:** The study used multiple algorithms to quantify the signal strength and then identified any previously unrecognized ADRs, further studies are needed to confirm the association of ADRs with eplerenone. The findings of this study may provide important insights into the safety profile of eplerenone, ensure that healthcare providers have up-to-date information about their potential risks and help guide them in the correct use of the drug.

## 1 Introduction

Eplerenone, an aldosterone antagonist, is used to increase survival in stable patients with symptomatic heart failure with reduced ejection fraction (HFrEF) following an acute myocardial infarction (FDA approved in 2002). Additionally, eplerenone has been shown to effectively reduce blood pressure and minimize the risk of both fatal and nonfatal cardiovascular events, particularly myocardial infarction and stroke. Dysregulated aldosterone activity can cause adverse effects on the cardiovascular system. By blocking the effects of aldosterone, it is possible to prevent some of these adverse cardiovascular effects and improve overall cardiovascular health (Sueta et al., 2020). At present, there are two aldosterone antagonists that are widely used in clinical practice: spironolactone and eplerenone (Buffolo et al., 2022). Spironolactone and aldosterone compete for binding to the mineralocorticoid receptor, which causes sodium and fluid retention (Rist et al., 2023). The mechanism of action for eplerenone is comparable (Tam et al., 2017; Agarwal et al., 2021). Compared to aldosterone, eplerenone has a shorter half-life, and it can be metabolized into highly inactive metabolites that are easier to flush out of the body. In clinical practice, the sexual side effect of spironolactone is the primary reason why hypertensive patients often struggle with medication adherence, but the use of eplerenone can significantly reduce the incidence of such adverse events (AEs) (Burgess, 2004; Struthers et al., 2008; Kolkhof and Bärfacker, 2017). In earlier clinical studies and randomized controlled trial, the most frequently reported ADRs of eplerenone for HFrEF were hyperkalaemia and blood creatinine increased, which were also listed in the prescribing information for eplerenone (Pitt et al., 2008; Provenzano et al., 2022). Fortunately, most AEs have not yet resulted in serious adverse consequences. However, due to the limited sample size and follow-up time, a large number of AEs are still not clearly reported on the label. Thus, a more extensive analysis of the safety of eplerenone is required (Dhillon, 2013; Ferreira et al., 2019).

The FDA uses the FDA Adverse Event Reporting System (FAERS) database to track AEs, medication errors and product quality complaints reported primarily by pharmaceutical companies. Using the data that drug companies provide to the FDA, it keeps tabs on the safety of pharmaceuticals and therapeutic biologics that are available on the market. The FAERS database compiles data that is provided by pharmaceutical companies, with the aim of monitoring the safety of drugs and therapeutic biologics on the market. It serves to identify potential safety signals and uncover latent safety concerns, thereby enhancing regulatory oversight and improving the safety of medications (Anand et al., 2019; Zhou and Hultgren, 2020). Recent research has shown that using the FAERS database to analyze medications can uncover a variety of potential negative effects associated with drugs. For example, poly (ADP-ribose) polymerase inhibitors (PARPis) are known as targeted drugs for cancer therapy, and many types of PARPis are widely used in clinical practice, but the differences between these PARPis have not been clarified in the real world. Using the information in the FAERS database, the differences in ADRs associated with different PARPis can be identified to improve the safety of the drug during treatment (Tian et al., 2022). Besides, quetiapine is an antipsychotic medication that has been approved for the treatment of various psychiatric diseases. Due to the limitations of clinical trials, we were unable to investigate the potential link between quetiapine and rare adverse cardiac events, but analysis using the FAERS database could offer valuable evidence for reducing the risk of quetiapine-related adverse cardiac events (Shu et al., 2023). Although the information in the FAERS database does not prove a direct causal relationship between the drug and AEs, it serves as a valuable resource for analyzing and monitoring drug safety.

In this study, four strict algorithms were used to screen eplerenone-related ADRs based on the FAERS database, comprehensively analyze the AEs in different systems, and explore the gender-based differences in AEs. Our study aims to identify and categorize additional AEs that aren’t listed on the label and provide recommendations for the safe clinical usage of eplerenone.

## 2 Materials and methods

### 2.1 Data source and collection

The FAERS database is the largest database in the world for reporting instances involving harmful drugs. It performs safety monitoring of therapeutic biological products and pharmaceuticals that have been put on the market by gathering safety information on them. Drug manufacturers are required to submit any collected reports to the FDA (https://www.fda.gov/drugs/questions-and-answers-fdas-adverse-event-reporting-system-faers/fda-adverse-event-reporting-system-faers-public-dashboard) (Omar et al., 2021; Zhang et al., 2022).

**FIGURE 1:**
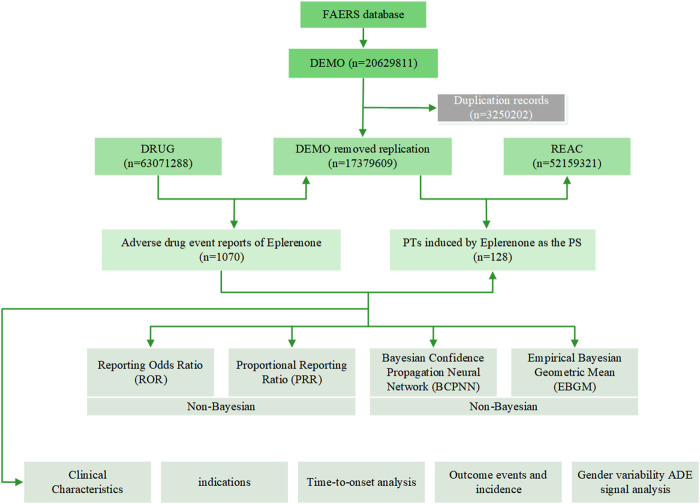
A flowchart of the study.

In order to improve accuracy, only eplerenone as PS drugs were retained in this study. In our study, we extracted eplerenone-related ADRs reported from 2004Q1 to 2023Q4 from the FAERS database. The data were captured and pre-processed using SAS and Navcat for MySQL software, and following straightforward processing, they were mapped to the MedDRA concept to eliminate duplicate case records and find AEs using statistical techniques, and then we computed significant AEs and mapped them to preferred terms (PTs) and system organ class (SOC) that correspond to various MedDRA levels (Figure 1). The study used the FAERS database to identify ADRs using eplerenone as the main suspected drug type. This study examined five factors: gender, weight, age, reported countries, and report source, with additional consideration of gender-based differences in ADRs.

### 2.2 Statistical analysis

The statistical analysis employed descriptive statistical methods to ADRs associated with eplerenone. To find pairs of ADRs associated with eplerenone, we employed several algorithms for metrics. We used four algorithms, including Reporting Odds Ratio (ROR) (Rothman et al., 2004), Proportional Reporting Ratio (PRR) (Evans et al., 2001), Bayesian Confidence Propagation Neural Network (BCPNN) (Ang et al., 2016) and Empirical Bayesian Geometric Mean (EBGM) (Rivkees and Szarfman, 2010) to calculate all AEs to find significant ADRs. Before computing the ROR and PRR, we initially obtained the four values of a, b, c and d (where a indicates total number of people who had the specified AEs after exposure to eplerenone, b indicates total number of people with non-target AEs after being exposed to eplerenone, c indicates total number of people with target AEs after exposure to non-eplerenone, d indicates total number of people who developed non-targeted AEs after being exposed to non-eplerenone (Table 1). These formulas are as follows:

**TABLE 1:** Table matrix.

	Eplerenone	Non-eplerenone
Target AEs	a	c
Non-target AEs	b	d
N = a + b + c + d		

#### 2.2.1 ROR algorithm

$$\text{ROR}=\frac{\text{ad}}{\text{bc}}$$

$${95\%\;\text{CI}=e}^{\ln\left(\text{ROR}\right)\pm 1.96\sqrt{\left(\frac{1}{a}+\frac{1}{b}+\frac{1}{c}+\frac{1}{d}\right)}}$$

If the lower limit of 95% CI > 1 and a ≥ 3, the ROR is a striking signal.

#### 2.2.2 PRR algorithm

$$\text{PRR}=\frac{a/\left(a+b\right)}{c/\left(c+d\right)}$$

$$\chi^{2}=\frac{{\left(\text{ad}-\text{bc}\right)}^{2}\left(a+b+c+d\right)}{\left(a+b\right)\left(a+c\right)\left(c+d\right)\left(b+d\right)}$$

If PRR ≥ 2, $\chi^{2}\geq$ 4, a ≥ 3 and *p* < 0.05 the PRR is a striking signal.

#### 2.2.3 BPCNN algorithm

$$\text{IC}=\log_{2}\frac{a\left(a+b+c+d\right)}{\left(a+b\right)\left(a+c\right)}$$

$$\text{IC}-2\text{SD}=E\left(\text{IC}\right)-2\sqrt{V\left(\text{IC}\right)}$$

If IC-2SD > 0 (IC-2SD: the lower bound of 95% CI), the BPCNN is a positive signal. The signal intensity was strikingly correlated with the IC-2SD value.

#### 2.2.4 EBGM algorithm

$$\text{EBGM}=\frac{a\left(a+b+c+d\right)}{\left(a+c\right)\left(a+b\right)}$$

$$95\%\text{ CI}=e^{\ln\left(\text{EBGM}\right)\pm 1.96\sqrt{\left(\frac{1}{a}+\frac{1}{b}+\frac{1}{c}+\frac{1}{d}\right)}}$$

If EBGM05 > 2 (EBGM05: the lower bound of 95% CI), the BPCNN is a striking signal.

We employed these four methods to find signals from the FAERS database from the first quarter of 2004 to the four quarter of 2023. The valid AE outcomes must concurrently satisfy the striking signal selection requirements in all four of the aforementioned methods. The data processing and statistical analysis in this study were conducted using SAS, MySQL, WPS EXCEL, and R software.

### 2.3 Gender-based differences of ADRs

In order to analyze the effect of gender on ADRs, we compared the ADRs of males and females after medication. Additionally, in order to evaluate the gender-specific risk of drug-related ADRs, ROR was utilized to determine the disproportional signals between males and females after eplerenone administration. If the ROR is greater than 1, it indicates that females have a higher risk for the ADR, to the contrary, if the ROR is less than 1, it indicates that males are at higher risk for the ADR.

## 3 Results

### 3.1 Descriptive results

From 2004Q1 to 2023Q4, a total of 20,629,811 reported cases were gathered from the FAERS database for this study. After being purged of duplicates and identified using four different techniques, 1,874 case reports were analyzed. Of these cases, 1,070 AEs were identified, 128 of which were eplerenone-related ADRs. Table 2 demonstrated the clinical features of eplerenone treatment events.

**TABLE 2:** The characters of case reports associated with eplerenone as primary suspected drug in FAERS (from 2004 Q1 to 2023 Q4).

Number of events	Counts 1874	Percentage (%)
**Gender**		
Male	1,136	60.60
Female	467	24.90
Unknown	271	14.50
**Weight (kg)**		
<50	38	2.00
50–100	483	25.80
>100	94	5.00
Unknown	1,259	67.20
**Age (years)**		
<18	2	0.10
18–64.9	427	22.80
65–85	804	42.90
>85	125	6.70
Unknown	516	27.50
**Reported countries (the top ranked)**		
US (United States of America)	279	14.90
JP (Japan)	277	14.80
FR (France)	213	11.40
GB (United Kiongdom)	178	9.50
DE (Germany)	136	7.30
**Report source (the top ranked)**		
Physician	747	39.90
Pharmacist	380	20.30
Consumer	340	18.10
Other health-professional	244	13.00
Health Professional	104	5.50

Among the selected case reports, there were 1,603 cases with specific gender information, 1,136 of these cases (60.6%) were male and 467 of them (24.9%) were female. Among the 615 case reports with clear weight information, patients weighing between 50 and 100 kg (25.8%) had a higher risk of AEs. However, due to the absence of specific weight information in the FAERS database, more studies are needed to confirm this result. The case reports mainly submitted by Physician (n = 747, 39.90%), Pharmacist (n = 380, 20.30%), Consumer (n = 340, 18.10%), Other health-professional (n = 244, 13.00%) and Health Professional (n = 104, 5.50%).

### 3.2 Signal detects at system organ class level

Supplementary Table S1 demonstrated the ADRs signal intensity of eplerenone at the SOC level. We discovered that eplerenone-related ADRs were observed in 27 systems, which suggested that eplerenone-related ADRs were relatively common. The top five SOCs were general disorders and administration site conditions (n = 747), investigations (n = 687), cardiac disorders (n = 485), renal and urinary disorders (n = 439), metabolism and nutrition disorders (n = 437). The SOCs that met at least one of the four algprithms included investigations (n = 747), renal and urinary disorders (n = 687), metabolism and nutrition disorders (n = 485), cardiac disorders (n = 439), reproductive system and breast disorders (n = 437), hepatobiliary disorders (n = 69), vascular disorders (n = 233), endocrine disorders (n = 33), ear and labyrinth disorders (n = 36) (Supplementary Table S2). There were three SOCs that met the four algorithms at the same time, namely: renal and urinary disorders (n = 439), metabolism and nutrition disorders (n = 437), cardiac disorders (n = 485), the side effects of these three systems could also be reflected in eplerenone’s drug label (Table 3).

**TABLE 3:** The signal strength of ADRs of eplerenone at the SOC level in the FAERS database (match four algprithms).

SOC name	Case numbers	ROR (95% CI)	PRR (95% CI)	Chi square	IC (IC025)	EBGM (EBGM05)
Cardiac Disorders	485	3.50 (3.19–3.84)	3.28 (788.76)	788.76	1.71 (0.05)	3.28 (3.03)
Renal And Urinary Disorders	439	4.35 (3.95–4.80)	4.09 (1,044.01)	1,044.01	2.03 (0.36)	4.09 (3.77)
Metabolism And Nutrition Disorders	437	3.84 (3.48–4.23)	3.61 (843.67)	843.67	1.85 (0.19)	3.61 (3.33)

### 3.3 Signal detections ranked by the EBGM at prefer terms level

During the study, four algorithms were employed to examine adverse medication reactions and assess whether they met the filtering criteria of the four algorithms. As illustrated in Table 4, the top 30 signals simultaneously recognized by the four algorithms in order of case numbers are presented, and all the results are shown in Supplementary Table S3. At the PTs level, there were 128 ADR terms related to 18 SOCs. The most rigorous algorithm, EBGM, was used to sort the 128 selected ADRs. The top five ADRs were: venous pressure jugular decreased (n = 3), hyperaldosteronism (n = 6), giardiasis (n = 4), hyperadrenocorticism (n = 4), N-Terminal prohormone brain natriuretic peptide increased (n = 11).

**TABLE 4:** The top 30 signal strength of ADRs of eplerenone at the PTs level recognized by the four algorithms simultaneously in FAERS, and ranked by case numbers.

PTs	SOC name	Case numbers	EBGM (EBGM05)
Acute Kidney Injury	Renal And Urinary Disorders	230	12.88 (11.53)
Hyperkalaemia	Metabolism And Nutrition Disorders	146	46.47 (40.49)
Hypotension	Vascular Disorders	92	5.03 (4.23)
Cardiac Failure	Cardiac Disorders	89	12.14 (10.19)
Drug Interaction	General Disorders And Administration Site Conditions	77	5.3 (4.39)
Hyponatraemia	Metabolism And Nutrition Disorders	68	13.12 (10.74)
Renal Impairment	Renal And Urinary Disorders	56	7.54 (6.05)
Renal Failure	Renal And Urinary Disorders	51	3.95 (3.13)
Dehydration	Metabolism And Nutrition Disorders	50	4.06 (3.22)
Oedema Peripheral	General Disorders And Administration Site Conditions	46	3.94 (3.09)
Blood Potassium Increased	Investigations	46	30.63 (24.02)
Blood Creatinine Increased	Investigations	42	6.86 (5.32)
Hypokalaemia	Metabolism And Nutrition Disorders	41	9.95 (7.69)
Atrial Fibrillation	Cardiac Disorders	38	4.26 (3.27)
General Physical Health Deterioration	General Disorders And Administration Site Conditions	37	3.87 (2.95)
Bradycardia	Cardiac Disorders	36	7.21 (5.48)
Blood Potassium Decreased	Investigations	29	10.48 (7.72)
Glomerular Filtration Rate Decreased	Investigations	29	29.44 (21.68)
Gynaecomastia	Reproductive System And Breast Disorders	24	7.49 (5.35)
Arrhythmia	Cardiac Disorders	24	5.42 (3.87)
Blood Pressure Decreased	Investigations	22	3.63 (2.56)
Cardiac Failure Chronic	Cardiac Disorders	21	55.58 (38.79)
Contraindicated Product Administered	Injury, Poisoning And Procedural Complications	21	7.83 (5.47)
Pulmonary Oedema	Respiratory, Thoracic And Mediastinal Disorders	19	4.59 (3.15)
Alanine Aminotransferase Increased	Investigations	19	3.3 (2.27)
Vertigo	Ear And Labyrinth Disorders	18	3.19 (2.17)
Product Prescribing Error	Injury, Poisoning And Procedural Complications	16	4.05 (2.69)
Ventricular Tachycardia	Cardiac Disorders	14	9.17 (5.91)
Polyuria	Renal And Urinary Disorders	14	18.56 (11.96)
Circulatory Collapse	Vascular Disorders	13	8.01 (5.08)

Among them, PT entries with more than 100 cases included acute kidney injury (n = 230), hyperkalaemia (n = 146), the second of which was consistent with the drug instructions published by FDA. Interestingly, we also found some AEs not mentioned in the instructions, which can have an effect on the respiratory system, including pulmonary oedema (n = 19), rales (n = 10); it can effect on the reproductive system and breast, including: gynaecomastia (n = 24), erectile dysfunction (n = 13); breast pain (n = 11); it may also affect the hepatobiliary system, including hepatic function abnormal (n = 11), alanine aminotransferase increased (n = 19), gamma-glutamyltransferase increased (n = 10). We also unexpectedly found that it may lead to rales (n = 18), ascites (n = 11) and gout (n = 11). In addition, using the EBGM, ROR, PRR and BCPNN screening algorithms alone, we were able to obtain 129, 183, 214, and 446 ADRs associated with eplerenone, respectively. These results are presented in Supplementary Tables S3–S6.

### 3.4 Gender-based differences of ADRs

To analyzed the gender-based differences in eplerenone-related ADRs, we used four algorithms to screen for drug-related ADRs in males and females. In males, we identified 101 drug-related ADRs (Supplementary Table S7), and in females, we identified 50 drug-related ADRs (Supplementary Table S8). Comparing the ADRs of different genders, we can find that 23 ADRs can be observed in both male and female, such as: hyperkalaemia, glomerular filtration rate decreased, acute kidney injury, etc. However, some ADRs can only be observed in certain genders. ADRs such as pemphigoid [n = 5, EBGM: 8.67 (4.16)], cutaneous vasculitis [n = 3, EBGM: 9.39 (3.64)], papule [n = 3, EBGM: 7.45 (2.89)], ear discomfort [n = 3, EBGM: 7.2 (2.79)], vertigo [n = 15, EBGM: 5.41 (3.54)], gout [n = 8, EBGM: 4.53 (2.53)] were only observed in males. ADRs such as haemorrhage subcutaneous [n = 3, EBGM: 39.65 (15.35)], hematochezia [n = 5, EBGM: 4.58 (2.2)], altered state of consciousness [n = 4, EBGM: 10.08 (4.43)] were only observed in females.

**FIGURE 2:**
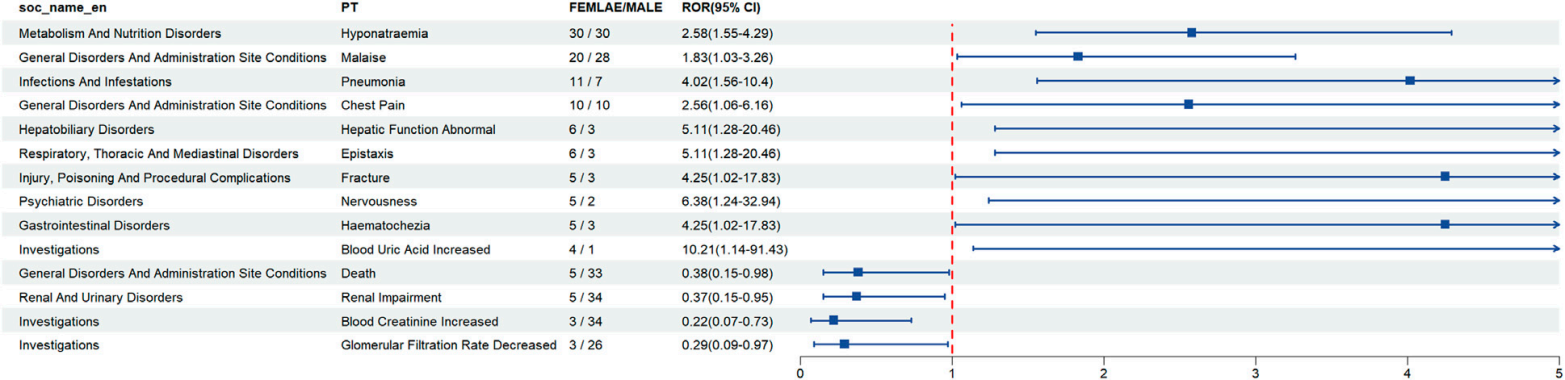
Analysis of gender-differentiated risk signals in eplerenone.

At the same time, ROR was used to assess the risk of gender-specific ADRs, and we found that ADRs such as hyponatraemia and chest Pain occur equally in males and females, malaise [1.83 (1.03–3.26)], death [0.38 (0.15–0.98)], renal impairment [0.37 (0.15–0.95)], blood creatinine increased [0.22 (0.07–0.73)], glomerular filtration rate decreased [0.29 (0.09–0.97)] were more likely to occur in males, pneumonia [4.02 (1.56–10.4)], hepatic function abnormal [5.11 (1.28–20.46)], epistaxis [5.11 (1.28–20.46)], fracture [4.25 (1.02–17.83)], nervousness [6.38 (1.24–32.94)], haematochezia [4.25 (1.02–17.83)], blood uric acid increased [10.21 (1.14–91.43)] were more likely to occur in females (Table 5; Figure 2).

**TABLE 5:** Analysis of gender-differentiated risk signals in eplerenone.

SOC	PT	Female/Male	ROR (95% CI)
Metabolism And Nutrition Disorders	Hyponatraemia	30/30	2.58 (1.55–4.29)
General Disorders And Administration Site Conditions	Chest Pain	10/10	2.56 (1.06–6.16)
General Disorders And Administration Site Conditions	Malaise	20/28	1.83 (1.03–3.26)
General Disorders And Administration Site Conditions	Death	5/33	0.38 (0.15–0.98)
Renal And Urinary Disorders	Renal Impairment	5/34	0.37 (0.15–0.95)
Investigations	Blood Creatinine Increased	3/34	0.22 (0.07–0.73)
Investigations	Glomerular Filtration Rate Decreased	3/26	0.29 (0.09–0.97)
Infections And Infestations	Pneumonia	11/7	4.02 (1.56–10.4)
Hepatobiliary Disorders	Hepatic Function Abnormal	6/3	5.11 (1.28–20.46)
Respiratory, Thoracic And Mediastinal Disorders	Epistaxis	6/3	5.11 (1.28–20.46)
Injury, Poisoning And Procedural Complications	Fracture	5/3	4.25 (1.02–17.83)
Psychiatric Disorders	Nervousness	5/2	6.38 (1.24–32.94)
Gastrointestinal Disorders	Haematochezia	5/3	4.25 (1.02–17.83)
Investigations	Blood Uric Acid Increased	4/1	10.21 (1.14–91.43)

## 4 Discussion

Cardiovascular disease (CVD) is the leading cause of death in western countries and poses a serious threat to human health (Farbehi et al., 2019; Hemanthakumar et al., 2021). CVD also imposes a substantial financial burden on patients (Patel et al., 2022). The GEO database serves as a rich repository of reports on CVD, offering a valuable resource for analyzing the underlying biological processes and identifying potential genetic targets for CVD treatment (Liu et al., 2021). Eplerenone has been approved by the FDA for the treatment of HFrEF since 2002. However, eplerenone has been relatively recently introduced into clinical practice, and it can be challenging to fully comprehend all the potential ADRs of eplerenone solely through clinical trials. Pre-marketing trials cannot generalize about all potential AEs in the real world. Therefore, it is necessary to continue to monitor the safety of drugs after marketing (Patel et al., 2022). In recent years, data mining using large databases as an exploratory research method for the prevention of ADRs has attracted people’s attention. FAERS database plays a significant function in the absence of clinical trials, especially when monitoring the long-term effects of drug therapies for chronic diseases like CVD becomes essential. The FAERS database gathers a variety of data on AEs and side effects associated with the usage of medicinal products, which is mostly submitted by drug manufacturers, with some data voluntarily submitted by drug users and other healthcare professionals (Yu et al., 2021; Yang et al., 2023). By accessing publicly available FAERS data, we can identify ADRs associated with eplerenone, monitor the drug’s safety, and provide guidance for its usage. In this study, we concentrated on AEs recorded in the FAERS database specifically for eplerenone, with the aim of identifying ADRs that were not recorded in the drug insert.

Significant SOCs identified by the four algorithms included cardiac disorders (n = 485), renal and urinary disorders (n = 439), metabolism and nutrition disorders (n = 437), which had been reported in clinical trials or shown in the drug label. ADRs mentioned in the FDA’s drug label include hyperkalemia, increased creatinine and hypertension. This was also confirmed in our study, which also illustrated the reliability of our results.

Four algorithms were used for analysis and screening. After excluding PT as the primary suspect (PS) for eplerenone therapy or complications, we concerned about some ADRs, which were not mentioned in the drug label, such as: glomerular filtration rate decreased [n = 29, EBGM: 29.44 (29.44)], creatinine renal clearance decreased [n = 11, EBGM: 27.62 (16.82)] and acute kidney injury [n = 230, EBGM: 12.88 (11.53)]. The drug label provided by the FDA noted that using eplerenone may increase creatinine levels, which indicated that eplerenone may cause impaired kidney function. Some studies have shown that treatment with eplerenone may increase the likelihood of a short-term decline in kidney function (Chung et al., 2020), however, eplerenone may also show renal protection in patients with normal or low aldosterone levels (Rafiq et al., 2011). Similar findings have been found in MRAs, which they suggest is haemodynamically mediated and reversible (at least up to > 30% change), which further indicates the potential renal benefits of eplerenone. This suggested that the effect of eplerenone on renal function may be related to baseline renal function. Therefore, renal function should be monitored in clinical applications.

Other ADRs we have noticed included giardiasis [n = 4, EBGM: 139.32 (60.97)] and *escherichia* sepsis [n = 3, EBGM: 11.21 (4.34)]. In one animal study, eplerenone treatment was found to promote the polarization of macrophages towards the M2-like phenotype (van der Heijden et al., 2018). However, the polarization of M1 macrophages will promote the phagocytosis of macrophages, and then kill colon *bacillus* (Chen et al., 2023). At the same time, the imbalance between gut microbiota and M2 macrophages can affect intestinal health and homeostasis, which may weaken the body’s protection against Giardia infection (Xaplanteri et al., 2023). Some studies have shown that eplerenone can inhibit ILC3, and then prevent the production of IL17, which has a protective effect against giardia infection (Xaplanteri et al., 2023; Zhao et al., 2024). These views were consistent with our findings. Although the number of reports of this ADR is not large, and it is not certain that this ADR is related to the use of eplerenone, we can not ignore the possible negative impact of eplerenone on the body’s immune mechanism. It is necessary to carry out further research to investigate the significance of this ADR and its association with eplerenone.

Among the screened ADRs, we also focused on gamma-glutamyltransferase increased [n = 10, EBGM: 4.67 (2.78)], alanine aminotransferase increased [n = 19, EBGM: 3.3 (2.27)] and hepatic function abnormal [n = 1q, EBGM: 3.4 (2.07)]. Although there are no reports of liver injury caused by eplerenone, a study reported in Clinical and Research Information on Drug-Induced Liver Injury suggested that eplerenone may be sensitive to acute liver injury (Eplerenone, 2012). The possible mechanism of liver injury is that the intermediates produced by the metabolism of eplerenone in the liver may cause liver reactions (Barnes and Howard, 2005). Therefore, in the course of medication, we should also pay attention to exclude potential liver injury.

In the reproductive system and breast diseases, we found that in addition to gynaecomastia, erectile dysfunction may also develop. In a study published in 2003, William F. Young Jr. reported that eplerenone is a selective aldosterone receptor blocker without the side effects associated with spironolides, including gynaecomastia, erectile dysfunction, and menstrual irregularities. However, our research showed that aldosterone receptor blockade may only partially reverse these effects, suggesting that eplerenone side effects may still exist (Lainscak et al., 2015). Therefore, these side effects should be closely concerned during the use of eplerenone, which may affect patient compliance.

According to our results, we also noticed some interesting ADRs, such as prothrombin time prolonged [n = 4, EBGM: 6.57 (2.89)] and activated partial thromboplastin time prolonged [n = 4, EBGM: 8.11 (3.57)]. We also unexpectedly found that the usage of eplerenone may increase blood uric acid (n = 5) and lead to gout (n = 11). Due to the small number and lack of control population, it is difficult to explain whether these ADRs are related to the use of eplerenone, but we should not ignore these possible ADRs when using eplerenone.

Based on the data in the baseline table, we could see that the incidence of ADRs in males is more than twice that in females. Therefore, gender differences must be considered when evaluating eplerenone-related ADRs. In order to further investigate the correlation between gender and ADRs, we further refined the gender-based subgroup analysis. From the results, we can observe that ear and labyrinth disorders, such as ear discomfort, vertigo, and infections and infestations disorders, such as giardiasis, *escherichia* sepsis, gastroenteritis were only observed in males. Renal And Urinary Disorders are more common in males, which may be due to differences in susceptibility to kidney disease between males and females (Ciarambino et al., 2022). Compared with males, the severity of ADRs in females is less, which may be due to the fact that females are more concerned about the abnormal performances of the body and actively seek medical treatment, while males are more inclined to not take intervention measures and wait for symptoms to resolve on their own, which may lead to further development of the disease (Lee et al., 2023). We noticed that the ADRs of the skin and subcutaneous tissue disorders were completely different in males and females, which may be due to the different sensitivity of the skin hair follicles to MR-mediated signaling in males and females (Hundt et al., 2019). These findings highlight the importance of paying attention to ADRs in patients of different genders in clinical practice. However, it is important to emphasize that more clinical evidence is needed to validate our findings.

Although this study identified many ADRs that are not currently of concern, there are inevitably some limitations to our study. Firstly, FAERS database is a spontaneous reporting database and there may be missing cases, so the database may not be complete and is not fully representative of real-world adverse reaction rates. Secondly, the drug-related ADRs obtained using these four algorithms only demonstrate that they are correlated, but whether there is a direct causal relationship between the drug and the adverse reaction requires further research. Thirdly, the FDA analyzes all collected adverse drug-related events, but it is difficult to determine a direct causal relationship with an exact drug that may be confounded by other interacting drugs. Finally, this study has only used the FAERS database for the analysis so far and should be validated with other databases to make the results more general.

It is essential to emphasize that prior to taking any prescribed medication, including eplerenone, we should read the instructions carefully to clarify the indications, contraindications and ADRs of the medication, and to comprehend how to correctly take it. Should any adverse reactions or side effects occur, it is advisable to seek immediate medical attention or consult with a healthcare professional.

## 5 Conclusion

Our study used data from the FAERS database to analyze the ADRs that occurred after the use of eplerenone. We have identified a number of ADRs that are not mentioned in the drug description, mainly manifested in four SOCs, including infections and infestations, renal and urinary disorders, reproductive system and breast disorders, and hepatobiliary disorders, but cohort studies and long-term clinical studies are still needed to validate these results and take us further to comprehend the relationship between eplerenone and these ADRs. This study will bring more attention to those adverse drug reactions that exist but have not yet received attention, which may provide recommendations for the subsequent use of the drug.

## Data Availability

Publicly available datasets were analyzed in this study. This data can be found here: https://www.fda.gov/drugs/questions-and-answers-fdas-adverse-event-reporting-system-faers/fda-adverse-event-reporting-system-faers-public-dashboard.

## Supplementary material

SUPPLEMENTARY TABLE S1 The signal strength of ADRs of eplerenone at the SOC level in the FAERS database.

SUPPLEMENTARY TABLE S2 The signal strength of ADRs of eplerenone at the SOC level in the FAERS database (match at least one of the four algorithms).

SUPPLEMENTARY TABLE S3 The signal strength of ADRs of eplerenone at the PTs level recognized by EBGM in FAERS.

SUPPLEMENTARY TABLE S4 The signal strength of ADRs of eplerenone at the PTs level recognized by ROR in FAERS.

SUPPLEMENTARY TABLE S5 The signal strength of ADRs of eplerenone at the PTs level recognized by PRR in FAERS.

SUPPLEMENTARY TABLE S6 The signal strength of ADRs of eplerenone at the PTs level recognized by BCPNN in FAERS.

SUPPLEMENTARY TABLE S7 The signal strength of ADRs of eplerenone in males (match four algorithms).

SUPPLEMENTARY TABLE S8 The signal strength of ADRs of eplerenone in females (match four algorithms).

## Abbreviations

ADR, adverse drug reaction; AE, adverse event; BCPNN, Bayesian Confidence Propagation Neural Network; CVD, Cardiovascular disease; EBGM, Empirical Bayesian Geometric Mean; FAERS, FDA Adverse Event Reporting System; HFrEF, heart failure with reduced ejection fraction; PARPis, poly(ADP-ribose) polymerase inhibitors; PRR, Proportional Reporting Ratio; PS, primary suspect; PT, preferred term; ROR, Reporting Odds Ratio; SOC, system organ class.

## References

[B1] Agarwal R. Kolkhof P. Bakris G. Bauersachs J. Haller H. Wada T. (2021). Steroidal and non-steroidal mineralocorticoid receptor antagonists in cardiorenal medicine. Eur. Heart J. 42 (2), 152–161. 10.1093/eurheartj/ehaa736 33099609 PMC7813624

[B2] Anand K. Ensor J. Trachtenberg B. Bernicker E. H. (2019). Osimertinib-induced cardiotoxicity: a retrospective review of the FDA adverse events reporting system (FAERS). JACC CardioOncol 1 (2), 172–178. 10.1016/j.jaccao.2019.10.006 34396179 PMC8352117

[B3] Ang P. S. Chen Z. Chan C. L. Tai B. C. (2016). Data mining spontaneous adverse drug event reports for safety signals in Singapore - a comparison of three different disproportionality measures. Expert Opin. Drug Saf. 15 (5), 583–590. 10.1517/14740338.2016.1167184 26996192

[B4] Barnes B. J. Howard P. A. (2005). Eplerenone: a selective aldosterone receptor antagonist for patients with heart failure. Ann. Pharmacother. 39 (1), 68–76. 10.1345/aph.1E306 15590870

[B5] Buffolo F. Tetti M. Mulatero P. Monticone S. (2022). Aldosterone as a mediator of cardiovascular damage. Hypertension 79 (9), 1899–1911. 10.1161/HYPERTENSIONAHA.122.17964 35766038

[B6] Burgess E. (2004). Eplerenone in hypertension. Expert Opin. Pharmacother. 5 (12), 2573–2581. 10.1517/14656566.5.12.2573 15571474

[B7] Chen X. Chen X. Yang Y. Luo N. Yang J. Zhong L. (2023). Protective role of the novel cytokine Metrnl/interleukin-41 in host immunity defense during sepsis by promoting macrophage recruitment and modulating Treg/Th17 immune cell balance. Clin. Immunol. 254, 109690. 10.1016/j.clim.2023.109690 37423488

[B8] Chung E. Y. Ruospo M. Natale P. Bolignano D. Navaneethan S. D. Palmer S. C. (2020). Aldosterone antagonists in addition to renin angiotensin system antagonists for preventing the progression of chronic kidney disease. Cochrane Database Syst. Rev. 10 (10), CD007004. 10.1002/14651858.CD007004.pub4 33107592 PMC8094274

[B9] Ciarambino T. Crispino P. Giordano M. (2022). Gender and renal insufficiency: opportunities for their therapeutic management? Cells 11 (23), 3820. 10.3390/cells11233820 36497080 PMC9740491

[B10] Dhillon S. (2013). Eplerenone: a review of its use in patients with chronic systolic heart failure and mild symptoms. Drugs 73 (13), 1451–1462. 10.1007/s40265-013-0098-z 23881669

[B11] Eplerenone (2012). LiverTox Clinical and research information on drug-induced liver injury. Bethesda (MD): National Institute of Diabetes and Digestive and Kidney Diseases. 31643176

[B12] Evans S. J. Waller P. C. Davis S. (2001). Use of proportional reporting ratios (PRRs) for signal generation from spontaneous adverse drug reaction reports. Pharmacoepidemiol Drug Saf. 10 (6), 483–486. 10.1002/pds.677 11828828

[B13] Farbehi N. Patrick R. Dorison A. Xaymardan M. Janbandhu V. Wystub-Lis K. (2019). Single-cell expression profiling reveals dynamic flux of cardiac stromal, vascular and immune cells in health and injury. ELife 8, e43882. 10.7554/eLife.43882 30912746 PMC6459677

[B14] Ferreira J. P. Abreu P. McMurray J. J. V. van Veldhuisen D. J. Swedberg K. Pocock S. J. (2019). Renal function stratified dose comparisons of eplerenone versus placebo in the EMPHASIS-HF trial. Eur. J. Heart Fail 21 (3), 345–351. 10.1002/ejhf.1400 30768732

[B15] Hemanthakumar K. A. Fang S. Anisimov A. Mäyränpää M. I. Mervaala E. Kivelä R. (2021). Cardiovascular disease risk factors induce mesenchymal features and senescence in mouse cardiac endothelial cells. ELife 10, e62678. 10.7554/eLife.62678 33661096 PMC8043751

[B16] Hundt J. E. Sass S. Funk W. Bíró T. Farman N. Langan E. A. (2019). Mineralocorticoid receptor antagonists stimulate human hair growth *ex vivo* . Skin. Pharmacol. Physiol. 32 (6), 344–348. 10.1159/000501729 31522177

[B17] Kolkhof P. Bärfacker L. (2017). 30 Years of the mineralocorticoid receptor: mineralocorticoid receptor antagonists: 60 years of research and development. J. Endocrinol. 234 (1), T125–T40. 10.1530/JOE-16-0600 28634268 PMC5488394

[B18] Lainscak M. Pelliccia F. Rosano G. Vitale C. Schiariti M. Greco C. (2015). Safety profile of mineralocorticoid receptor antagonists: spironolactone and eplerenone. Int. J. Cardiol. 200, 25–29. 10.1016/j.ijcard.2015.05.127 26404748

[B19] Lee K. M. N. Rushovich T. Gompers A. Boulicault M. Worthington S. Lockhart J. W. (2023). A Gender Hypothesis of sex disparities in adverse drug events. Soc. Sci. Med. 339, 116385. 10.1016/j.socscimed.2023.116385 37952268

[B20] Liu C. Chen S. Zhang H. Chen Y. Gao Q. Chen Z. (2021). Bioinformatic analysis for potential biological processes and key targets of heart failure-related stroke. J. Zhejiang Univ. Sci. B 22 (9), 718–732. 10.1631/jzus.B2000544 34514752 PMC8435344

[B21] Omar N. E. Fahmy Soliman A. I. Eshra M. Saeed T. Hamad A. Abou-Ali A. (2021). Postmarketing safety of anaplastic lymphoma kinase (ALK) inhibitors: an analysis of the FDA Adverse Event Reporting System (FAERS). ESMO Open 6 (6), 100315. 10.1016/j.esmoop.2021.100315 34864500 PMC8649649

[B22] Patel N. M. Stottlemyer B. A. Gray M. P. Boyce R. D. Kane-Gill S. L. (2022). A pharmacovigilance study of adverse drug reactions reported for cardiovascular disease medications approved between 2012 and 2017 in the United States food and drug administration adverse event reporting system (FAERS) database. Cardiovasc. Drugs Ther. 36 (2), 309–322. 10.1007/s10557-021-07157-3 33599896

[B23] Pitt B. Bakris G. Ruilope L. M. DiCarlo L. Mukherjee R. EPHESUS Investigators (2008). Serum potassium and clinical outcomes in the eplerenone post-acute myocardial infarction heart failure efficacy and survival study (EPHESUS). Circulation 118 (16), 1643–1650. 10.1161/CIRCULATIONAHA.108.778811 18824643

[B24] Provenzano M. Puchades M. J. Garofalo C. Jongs N. D’Marco L. Andreucci M. (2022). Albuminuria-lowering effect of dapagliflozin, eplerenone, and their combination in patients with chronic kidney disease: a randomized crossover clinical trial. J. Am. Soc. Nephrol. 33 (8), 1569–1580. 10.1681/ASN.2022020207 35440501 PMC9342643

[B25] Rafiq K. Nakano D. Ihara G. Hitomi H. Fujisawa Y. Ohashi N. (2011). Effects of mineralocorticoid receptor blockade on glucocorticoid-induced renal injury in adrenalectomized rats. J. Hypertens. 29 (2), 290–298. 10.1097/hjh.0b013e32834103a9 21243738 PMC3034279

[B26] Rist A. Sevre K. Wachtell K. Devereux R. B. Aurigemma G. P. Smiseth O. A. (2023). The current best drug treatment for hypertensive heart failure with preserved ejection fraction. Eur. J. Intern. Med. 120, 3–10. 10.1016/j.ejim.2023.10.008 37865559

[B27] Rivkees S. A. Szarfman A. (2010). Dissimilar hepatotoxicity profiles of propylthiouracil and methimazole in children. J. Clin. Endocrinol. Metab. 95 (7), 3260–3267. 10.1210/jc.2009-2546 20427502

[B28] Rothman K. J. Lanes S. Sacks S. T. (2004). The reporting odds ratio and its advantages over the proportional reporting ratio. Pharmacoepidemiol Drug Saf. 13 (8), 519–523. 10.1002/pds.1001 15317031

[B29] Shu Y. Ding Y. Liu L. Zhang Q. (2023). Cardiac adverse events associated with quetiapine: disproportionality analysis of FDA adverse event reporting system. CNS Neurosci. Ther. 29, 2705–2716. 10.1111/cns.14215 37032639 PMC10401141

[B30] Struthers A. Krum H. Williams G. H. (2008). A comparison of the aldosterone-blocking agents eplerenone and spironolactone. Clin. Cardiol. 31 (4), 153–158. 10.1002/clc.20324 18404673 PMC6652937

[B31] Sueta D. Yamamoto E. Tsujita K. (2020). Mineralocorticoid receptor blockers: novel selective nonsteroidal mineralocorticoid receptor antagonists. Curr. Hypertens. Rep. 22 (3), 21. 10.1007/s11906-020-1023-y 32114686

[B32] Tam T. S. Wu M. H. Masson S. C. Tsang M. P. Stabler S. N. Kinkade A. (2017). Eplerenone for hypertension. Cochrane Database Syst. Rev. 2 (2), CD008996. 10.1002/14651858.CD008996.pub2 28245343 PMC6464701

[B33] Tian X. Chen L. Gai D. He S. Jiang X. Zhang N. (2022). Adverse event profiles of PARP inhibitors: analysis of spontaneous reports submitted to FAERS. Front. Pharmacol. 13, 851246. 10.3389/fphar.2022.851246 35401230 PMC8990839

[B34] van der Heijden C. D. C. C. Deinum J. Joosten L. A. B. Netea M. G. Riksen N. P. (2018). The mineralocorticoid receptor as a modulator of innate immunity and atherosclerosis. Cardiovasc Res. 114 (7), 944–953. 10.1093/cvr/cvy092 29668907

[B35] Xaplanteri P. Rodis N. Potsios C. (2023). Gut microbiota crosstalk with resident macrophages and their role in invasive amebic colitis and giardiasis-review. Microorganisms 11 (5), 1203. 10.3390/microorganisms11051203 37317178 PMC10221053

[B36] Yang H. Wan Z. Chen M. Zhang X. Cui W. Zhao B. (2023). A real-world data analysis of topotecan in the FDA Adverse Event Reporting System (FAERS) database. Expert Opin. Drug Metabolism Toxicol. 19, 217–223. 10.1080/17425255.2023.2219390 37243615

[B37] Yu R. J. Krantz M. S. Phillips E. J. Stone C. A. (2021). Emerging causes of drug-induced anaphylaxis: a review of anaphylaxis-associated reports in the FDA adverse event reporting system (FAERS). J. Allergy Clin. Immunol. Pract. 9 (2), 819–829.e2. 10.1016/j.jaip.2020.09.021 32992044 PMC7870524

[B38] Zhang P. Lao D. Chen H. Zhao B. Du Q. Zhai Q. (2022). Neuromuscular junction dysfunctions due to immune checkpoint inhibitors therapy: an analysis of FAERS data in the past 15 years. Front. Immunol. 13, 778635. 10.3389/fimmu.2022.778635 36081514 PMC9446345

[B39] Zhao R. Hong L. Shi G. Ye H. Lou X. Zhou X. (2024). Mineralocorticoid promotes intestinal inflammation through receptor dependent IL17 production in ILC3s. Int. Immunopharmacol. 130, 111678. 10.1016/j.intimp.2024.111678 38368773

[B40] Zhou Z. Hultgren K. E. (2020). Complementing the US food and drug administration adverse event reporting system with adverse drug reaction reporting from social media: comparative analysis. JMIR Public Health Surveillance 6 (3), e19266. 10.2196/19266 32996889 PMC7557434

